# What happens to regulatory T cells in multiple myeloma

**DOI:** 10.1038/s41420-023-01765-8

**Published:** 2023-12-21

**Authors:** Huixian Chen, Xueling Wang, Yan Wang, Xiaotian Chang

**Affiliations:** 1 Medical Research Center, The Affiliated Hospital of Qingdao University, Qingdao, 266003 China; 2 Department of Hematology, The Affiliated Hospital of Qingdao University, Qingdao, 266003 China; 3 Department of Gastroenterology, The Affiliated Hospital of Qingdao University, Qingdao, 266003 China; 4 Department of Pediatrics, Qilu Hospital of Shandong University, Jinan, 250012 China

**Keywords:** Cancer microenvironment, Myeloma

## Abstract

Abnormal tumor microenvironment and immune escape in multiple myeloma (MM) are associated with regulatory T cells (Tregs), which play an important role in maintaining self-tolerance and regulating the overall immune response to infection or tumor cells. In patients with MM, there are abnormalities in the number, function and distribution of Tregs, and these abnormalities may be related to the disease stage, risk grade and prognosis of patients. During the treatment, Tregs have different responses to various treatment regiments, thus affecting the therapeutic effect of MM. It is also possible to predict the therapeutic response by observing the changes of Tregs. In addition to the above, we reviewed the application of Tregs in the treatment of MM. In conclusion, there is still much room for research on the mechanism and application of Tregs in MM.

## Facts

1. The progression, recurrence and drug resistance of multiple myeloma are related to its inhibitory immune microenvironment.
2. Regulatory T cells play an important role in maintaining self-tolerance and regulating the overall immune response to infection or tumor cells, and can inhibit the activation, proliferation and function of a variety of immunocompetent cells.
3. Regulatory T cells have been shown to have massive infiltration of a variety of solid tumors in humans and mice as well as increased cellular levels in the peripheral blood.

## Questions

1. What changes occur in the number and function of Treg cells in patients with multiple myeloma?
2. Is there a correlation between the frequency of Treg cells and disease severity or prognosis in patients with multiple myeloma?
3. What are the effects of drugs on Treg cells in multiple myeloma, and what are the applications of treg cells in therapy?

## Introduction

Multiple myeloma (MM) is the most common form of plasma cell malignancy, accounting for about 1.7% of all malignant tumors [1]. Although the use of a variety of treatment options such as high-dose chemotherapy, hematopoietic stem cell transplantation (HSCT), proteasome inhibitors (PIs), immunomodulatory drugs

(IMiDs) and monoclonal antibodys (mAbs) has greatly improved remission rates in MM [2], it remains an incurable disease. Almost all patients relapse and progress after initial treatment.

## Tumor microenvironment in multiple myeloma

The abnormality of tumor microenvironment (TME) and immune escape, as the causes of tumor recurrence and progression, have been paid more and more attention [3, 4]. In MM, the inhibition of normal polyclonal immunoglobulin secretion results in humoral immune dysfunction. Activation receptors on the surface of cytotoxic T lymphocytes and natural killer (NK) cells are down-regulated, resulting in functional deficits [5, 6]. At the same time, malignant plasma cells block antigen presentation by increasing the expression of inhibitory receptor ligands [7, 8], decreasing or decomposing the expression of activator receptor ligands, down-regulating MHC I genes, or altering tumor-related epitopes [9–11], so as to escape the killing effect of immune cells. In addition, myeloma cells can change the chemokine microenvironment to promote the migration of NK cells out of the bone marrow and the development and recruitment of immunosuppressive cells, such as myeloid-derived suppressor cells (MDSCs), tumor-associated macrophages (TAMs), regulatory B cells (Bregs), and regulatory T cells (Tregs) [12–14]. Multiple mechanisms work together to cause the MM tumor growth and breakdown of immune control.

## Regulatory T cells

### Classification of regulatory T cells

Tregs are a subset of mature T cells with regulatory function, which play an important role in maintaining self-tolerance and regulating the overall immune response to infection or tumor cells. Tregs can inhibit the activation, proliferation and function of immune cells such as CD4^+^ and CD8^+^ T cells, NK cells, B cells and antigen presenting cells (APCs). There are different Treg cell types in human body, including CD4^+^, CD8^+^ and CD4^-^CD8^-^ double negative Tregs [15]. CD4^+^ Tregs can suppress immunity in a non-specific manner, accounting for ~5–10% of the peripheral blood CD4^+^ T cells [16], which is also the main discussion object in this paper. CD4^+^ Tregs can be divided into native Tregs (nTregs) and peripheral Tregs (iTregs) [17]. The former develops from thymocytes, functions mainly through cell contact mechanism, and has stable immunosuppressive function [18, 19]. The latter is produced by naive T cells in response to persistent antigen stimulation and cytokines such as IL-2 and TGF-β, and can also be induced by cell-cell contact [20]. iTregs, which are mainly found in mucosal sites and chronic inflammatory tissues [21, 22], function by releasing soluble cytokines and have the potential to redifferentiate into Th17 cells and express IL-17 [23, 24].

### Molecules associated with regulatory T cells

The immunosuppressive function of Tregs depends on continuous expression of the transcription factor Foxp3, which is also a hallmark of the completion of Tregs differentiation [25]. Foxp3 controls the proliferation, inhibitory function and homeostasis of Treg cells by regulating oxidative phosphorylation [26, 27]. Foxp3 can regulate the expression level of cytotoxic t lymphocyte-associated protein 4(CTLA-4) on the surface of Treg cells, and there is a linear correlation between them [28, 29]. Mutations of Foxp3 gene can hinder the development of Tregs and lead to a fatal multi-organ autoimmune disease which is called X-linked polyendocrinopathy enteropathy with immune dysregulation syndrome [30]. In addition, CD4^+^ T cells can also transiently express Foxp3 upon activation [31].

Before the discovery of Foxp3, Tregs were often identified as CD4^+^CD25^+^. CD25 is the α chain of IL-2 receptor (IL-2R). Foxp3 induction in the thymus and homeostasis of Tregs depend on its signaling: IL-2 binding to IL-2R leads to phosphorylation of STAT5, and phosphorylated STAT5 binds to the Foxp3 promoter to increase Foxp3 transcription and expression levels [32]. However, Tregs rarely produce IL-2 and are highly dependent on exogenous IL-2 [33]. Low dose of IL-2 can promote the expansion of Treg by enhancing the expression of anti-apoptotic protein BCL-2 [34]. Application of IL-2 neutralizing antibody to CD25 can reduce the number of Foxp3^+^ Treg cells and the expression of Foxp3 in individual cell, and even induce the occurrence of autoimmune diseases [19, 35]. Furthermore, CD25 is transiently expressed on activated CD8^+^ effector T cells, which, when combined with IL-2, can effectively activate T cells and promote T cell proliferation. However, when CD25 on the surface of Treg cells competitively binds to IL-2, the proliferation of effector T cells can be inhibited.

CD127 is a type I transmembrane glycoprotein also known as IL-7 receptor α chain (IL-7Rα). Foxp3 binds to the promoter of CD127, and its unique forkhead DNA-binding domain is close to the C terminus of the protein, which inhibits the transcription of CD127 [36]. Seddiki et al. also found that human Tregs consistently express lower levels of CD127 compared to most other CD4^+^ T cells and that Foxp3 expression was inversely correlated with CD127 in CD4^+^CD25^+^ T cells [37]. Interestingly, it has been proposed that the expression profile of CD127 on Tregs depends on its tissue localization. Some Treg cells may show an increase in CD127 expression after activation and this was demonstrated in a model of skin inflammation [38].

Klein et al. [39] tried to simultaneously use CD4^+^CD25^+^CD127^-/low^ and CD4^+^CD25^+^Foxp3^+^ to define Tregs in neonatal peripheral blood and found that they represented two distinct and intersecting cell subsets. The correlation between Foxp3 and Treg phenotype was stronger. In addition, the differentiation of CD127^+^ and CD127^-/low^ cells was not obvious in flow cytometry analysis, which made it more difficult to define the cells. Some researchers believed that the Tregs obtained by the two gating methods of CD25^+^Foxp3^+^ or CD25^+^CD127^-/low^ in healthy people were identical. But in patients with suspected immunodeficiency, it was suggested to use CD25^+^CD127^-/low^ and CD25^+^Foxp3^+^ in combination to detect Tregs to avoid false positive results [40]. Żabińska et al. [41] also proposed that gating with CD4^+^CD25^+^CD127^-/low^ should be used with caution in autoimmune-related diseases. However, the detection of Foxp3 requires permeabilization of the cell membrane, which irreversibly impairs cell viability and cannot be used to collect active Treg cells. In general, the gating method of Tregs needs to be selected according to the actual situation.

**Fig. 1:**
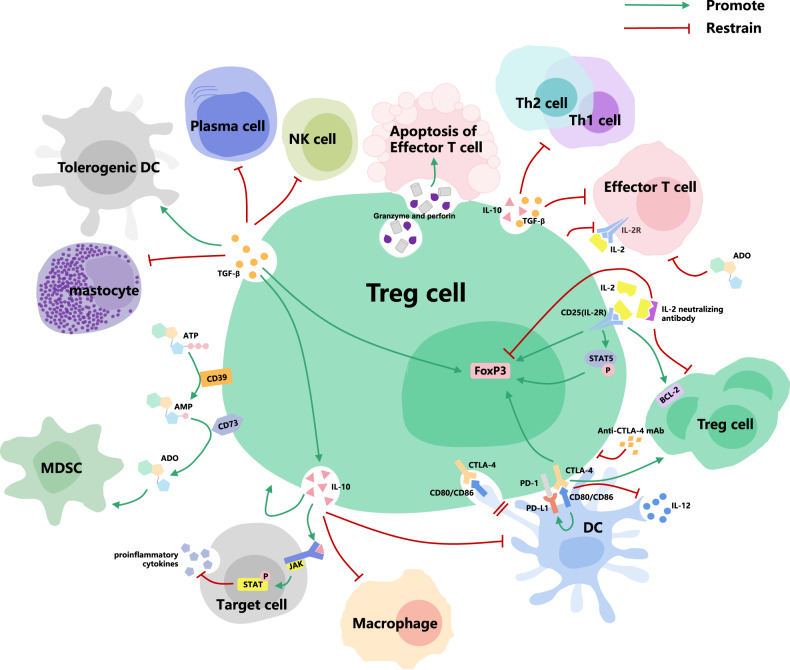
Mechanism of regulatory T cells. Tregs down-regulate the expression of CD80/CD86 in DCs through CTLA-4, increase the expression of PD-L1, promote the binding of Treg-DC, reduce the secretion of IL-12, and hinder the antigen presentation.Tregs express IL-2 receptor, which competitively binds to IL-2 to increase Foxp3 transcription and inhibit Treg cell apoptosis and effector T cell proliferation. Tregs can also secrete IL-10 and TGF-β to inhibit the function of immunocompetent cells such as DCs, macrophages, mastocytes, Th1/Th2 cells, cytotoxic T cells, NK cells, and plasma cells. Granzyme is expressed by Tregs and kills effector T cells through perforin-dependent cytotoxicity on the basis of direct cell contact. In addition, Tregs inhibit effector T cell responses and induce MDSCs via the CD39/CD73 adenosine pathway.

In addition to the above markers of Treg cells, there are many other molecules involved in their immunosuppressive function. CTLA-4 is a negative regulator of T cell activation that shares the molecular ligand CD80/CD86 with CD28 and has a higher affinity [42]. CD80/CD86 is expressed on mature dendritic cells (DCs). Tregs can down-regulate the expression of CD80/CD86 on DCs through CTLA-4-dependent trogocytosis, increase the expression of free PD-L1, promote the binding of Tregs-DCs and the formation of immune synapse, thereby interfere with antigen-presenting function of DCs and reduce the secretion of pro-inflammatory factor IL-12. Meanwhile, the proliferation of treg cells and the expression of Foxp3 protein were promoted [43–45]. Anti-CTLA-4 treatment can inhibit Tregs function [46]. IL-10 is an immunosuppressive factor that is simultaneously expressed in many cells of the adaptive and innate immune systems [47]. IL-10 inhibits the production of proinflammatory cytokines by target cells through activation of JAK-STAT signaling pathway and acts on DCs or macrophages to inhibit the development of Th1-type responses. It also inhibits Th2 cells and allergic responses. Tregs are an important source of IL-10 in several organs such as intestine, lung, skin and central nervous system, and control immune homeostasis through IL-10 production [48, 49]. At the same time, IL-10 enhances the differentiation of Treg cells through a positive regulatory circuit, helping to maintain their phenotype and function [50]. In addition, activated Treg cells can secrete TGF-β. TGF-β can inhibit the proliferation and function of immunocompetent cells, induce immature DCs to become tolerogenic DCs, and inhibit the production of pro-inflammatory cytokines [51]. Allergic and autoimmune responses can be limited by TGF-β. In mice susceptible to food allergy, reduced TGF-β1 expression in Tregs can be observed, and active knockout of TGF-β1 alleles in Tregs can enhance allergic response [52]. TGF-β can induce the expression of Foxp3 to promote the generation of Treg cells, which transforms peripheral blood CD4^+^ naïve T cells into CD4^+^CD25^+^Foxp3^+^ Tregs [53]. The production of IL-10 by Tregs also depends on the presence of TGF-β [54]. Moreover, activated Tregs can also express granzyme, kill target cells through perforin-dependent cytotoxicity and induce effector T cell exhaustion on the basis of direct contact between cells [55]. Another important mechanism of Treg cell-mediated suppression involves the CD39/CD73 adenosine (ADO) pathway. Tregs hydrolyse extracellular ATP and produce ADO through CD39/CD73. Binding of extracellular ADO to its cognate receptor inhibits effector T-cell responses and induces MDSCs to exert their effects [56]. The above mechanisms related to Tregs are shown in Fig. 1.

### Regulatory T cells and diseases

Studies have shown that Tregs promote the formation of an immunosuppressive environment by secreting cytokines, and their interactions with stromal cells such as fibroblasts and endothelial cells support the survival of cancer cells [57]. Treg cells, which are specifically recruited by chemotactic effect, have been shown to have massive infiltration of a variety of solid tumors in humans and mice as well as increased cellular levels in the peripheral blood [58–61]. Immunosuppressive function of tumor-induced Tregs is stronger [62] and significantly negatively correlated with patient survival [63]. It has also been proposed that Tregs are selectively aggregated in tumors and that their frequency in peripheral blood does not correctly reflect the tumor microenvironment [64]. Tregs in hematological malignancies have also been explored. Studies have shown that in the early stage of myelodysplastic syndrome(MDS), treg cells show a deficiency in immunosuppressive function, and in the advanced stage of the disease, their function is restored and their number is enlarged [65, 66]. CD4^+^Tregs are associated with higher risk and progression of MDS [67]. Recruitment of Treg cells in peripheral blood and bone marrow was also found in acute or chronic lymphocytic leukemia and myeloid leukemia [68–71]. In 1976, Waldmann et al. proposed for the first time that one of the causes of humoral immunity defects in MM may be abnormal or prolonged presence of regulatory T cells [72].

## Regulatory T cells in multiple myeloma

### Number and function of regulatory T cells in multiple myeloma

The transformations from monoclonal gammopathy of unknown significance (MGUS) and smoldering multiple myeloma (SMM) to MM progression are related to sequence gene mutations, but also to the significant changes in the cell composition of the bone marrow microenvironment. The inhibitory immune microenvironment is also the basis of drug resistance and disease recurrence [73]. Although the expansion and role of Treg cells have been demonstrated in a variety of tumors, many different insights exist in MM (Table 1) [74].

**Table 1:** Regulatory T cells in multiple myeloma (all compared to healthy controls).

Year	Study	MGUS				MM					Gating method	Reference
		Peripheral blood		Bone marrow		Peripheral blood		Bone marrow		Subject		
		Number	Function	Number	Function	Number	Function	Number	Function			
2006	Prabhala RH	↓	↓			↓	↓			NDMM	CD4+Foxp3+	[82]
2006	Beyer M	↑	ns	↑	ns	↑	ns	↑	ns	MM^*^	CD4^+^CD25^high^Foxp3^+^	[86]
2008	Laronne-Bar-On A					↑	ns	↑^†^		mouse model	CD4^+^CD25^high^Foxp3^+^	[80]
2009	Feyler S	**↑**				↑	ns	ns		NDMM/LDMM/RRMM	CD4^+^CD25^+^Foxp3^+^ (nTreg)	[87]
		↓				↓		ns			CD3+CD4-CD8-abTCR+ (DN Treg)	
2010	Noonan K					↑		↓		MM	CD4^+^CD25^+^Foxp3^+^	[99]
2010	Brimnes MK	ns				↑	ns			NDMM	CD4+Foxp3+	[83]
						ns	ns			LDMM		
2011	Gupta R					↓	ns			NDMM	CD4+CD25+CD127dimFoxp3+	[90]
2012	Muthu Raja KR	ns				↑	ns			NDMM	CD4^+^CD25^high^Foxp3^+^	[102]
						ns				LDMM		
						↑				RRMM		
2012	Muthu Raja KR					↑	ns			NDMM	CD8+CD25highFoxp3+	[88]
2012	Giannopoulos K					↑				NDMM	CD4+CD25highFoxp3+	[91]
2013	Bryant C					↑				LTS-MM	CD3+CD4+CD25++CD127-	[104]
2014	Raja KR					↑				NDMM	CD8+CD25highFoxp3+	[116]
2014	Fu R							↑		NDMM	CD4+CD25+CD127low	[95]
2014	Foglietta M			ns		ns	ns	ns	ns	NDMM/LDMM/RRMM	CD4+CD25highFoxp3+/CD4+CD25+CD127lowFoxp3+	[96]
2014	Braga WM							↑		NDMM	CD3+CD4+CD25highFoxp3+CTLA4+	[29]
2015	Frassanito MA					↑				NDMM	CD4+Foxp3+	[77]
						↑				LDMM		
						↑				NDMM	CD8+Foxp3+	
						ns				LDMM		
2015	Feng P					↓				MM	CD4+CD25+Foxp3+	[89]
2016	Paiva B					↑				high-risk SMM		[105]
2016	Ma Y					↑	ns			NDMM/LDMM/RRMM	TCRγδ+Foxp3+CD27+CD25high	[92]
2016	D’Arena G	ns				ns				NDMM	CD4highCD25highCD127-/low	[97]
2017	Feng X					↑				MM	CD4+CD25highFoxp3+	[20]
2017	Aref S					↑				NDMM		[103]
2018	Wang JN	aTreg ↑		aTreg ↑		aTreg ↑		aTreg ↑	ns	NDMM	CD4+Foxp3+	[85]
		rTreg ns		rTreg ↓		rTreg ns		rTreg ↓				
		non-Treg ns		non-Treg ns		non-Treg ns		non-Treg ns				
2018	Kawano Y					↑		↑		mouse model	CD4+Foxp3+	[14]
								↑		SMM	CD4+CD25+Foxp3+/CD4+CD25+Foxp3+CD127-/low	
2019	Vela-Ojeda J					ns				NDMM	CD4+CD25+Foxp3+	[98]
2019	Marsh-Wakefield F	ns		ns		ns		ns		NDMM	CD4+CD25+CD127-/low	[107]
2019	Lad D			ns		↑	ns	ns		NDMM	CD4+CD25highCD127low	[106]
						↑					CD45RA-CCR7-Ki67high (activated or effector Treg)	
						↑					CD45RA-CCR7-Ki67low (memory Treg)	
2020	Alrasheed N							↑	↑	NDMM	CD4+Foxp3+/CD4+Foxp3+CD25+	[94]
2021	Kulikowska de Nałęcz A					↑				NDMM/RRMM	CD4+CD25+CD127-/CD4+CD25+Foxp3+/CD4+Foxp3+CD127^-^	[93]
2022	Bae J	ns		ns		↑		ns		NDMM	CD4+CD25+Foxp3+	[136]
						↑		↑		RRMM		
						ns		ns		SMM		

*MGUS* monoclonal gammopathy of uncertain significance, *MM* multiple myeloma, *NDMM* newly diagnosed patients, *LDMM* patients with low disease burden/plateau, *RRMM* relapsed/refractory patients, *LTS-MM* patients with long-term survival, *SMM* smoking multiple myeloma, *↑* Increased number or enhanced functionality, *↓* reduced quantity or inhibited function, *ns* no significant difference.

† In the progressive stages of the disease.

*NDMM or patients who had not received cytoreductive treatment for a period of at least 1 month prior to investigation.

#### Cell models and animal models

In vitro studies have shown that MM cells can induce IL-10 production by nTreg cells and promote significant proliferation of CD4^+^CD25^+^Foxp3^+^ iTreg cells in a contact-dependent manner, showing increased expression of Foxp3, GITR, PD1 and CD62L compared with nTreg cells [75, 76]. MM cells can also induce Tregs by converting Foxp3^-^ to Foxp3^+^ cells [77]. Bone marrow stromal cells (BMSCs) can increase quantity and activity of Tregs [78]. Co-culture of myeloma derived exosomes with healthy Treg cells can inhibit the apoptosis of the latter [79]. In the MM mouse model, a significant increase in Tregs could be observed in the spleen and lymph nodes, in contrast, Tregs’ frequency in the bone marrow remained normal in the early stage and increased significantly only at the stage of disease progression [80]. However, in one study, immunofluorescence staining of bone marrow sections of MM mice showed that Treg cells were highly accumulated at the tumor location, and expressed higher levels of CD25, ICAM-1, CD69, CD44 and co-inhibitory receptors Lag-3, Tim-3, TIGIT and PD-1 [81].

#### Changes in treg cells in patients

In a study published in *Blood* in 2006, Prabhala et al. [82] used CD4^+^Foxp3^+^ gating to detect the proportion of Tregs in PBMC from healthy people, MGUS and MM patients, and evaluated their ability to inhibit T cell proliferation. The results showed that Tregs in the peripheral blood of MGUS and MM patients were reduced and had a loss of function. Whether these cells co-express CD25 was not determined in this study. Another study also used CD4^+^Foxp3^+^ as gating, and proved that the proportion of Treg cells in peripheral blood increased in newly diagnosed MM, but there was no significant change in remission stage and MGUS [83]. Foxp3^+^CD4^+^ T cells can be divided into three subsets according to the expression levels of CD45 and Foxp3: CD45RA^+^Foxp3^low^ quiescent naive Treg cells (rTregs), CD45RA^-^Foxp3^high^ activated Treg cells (aTregs) and CD45RA^-^Foxp3^low^ non-Treg cells (non-Tregs) [84]. In a study published in 2018, these three subsets were studied [85]. The results showed that in peripheral blood, the levels of aTregs in MGUS and MM were significantly increased, while the frequencies of rTregs and non-Tregs were not significantly different. In the bone marrow, the frequency of aTregs was also higher in MGUS and MM than in healthy controls, and there was no significant difference in non-Tregs. But the frequency of rTregs was significantly reduced. There was no significant difference in the levels of aTregs in the peripheral and bone marrow between MGUS and MM patients. And the inhibitory ability of the three Treg cell subsets in MM was not different from that in healthy controls. These results once again illustrate the importance of discussing the heterogeneity of CD4^+^Foxp3^+^ cells. Beyer et al. [86] identified CD4^+^CD25^high^Foxp3^+^ Tregs from MM patients and evaluated suppressive function using a modified allogeneic mixed lymphocyte reaction(MLR). The results showed that the frequency of CD4^+^CD25^high^Foxp3^+^ Tregs increased in the peripheral blood and bone marrow of MGUS and MM patients, and the suppressive function was normal after the number standardization. There was no significant difference in Tregs level between patients with or without treatment. In addition, in MGUS and MM, Tregs expressed higher levels of Foxp3, CTLA4, GITR and CD62L, and naïve Tregs were significantly expanded. Feyler et al. [87] for the first time evaluated the differences in levels and functions of different types of Tregs in MM and discussed them according to disease stage. The results showed that functional CD4^+^CD25^+^Foxp3^+^ nTregs were significantly increased in peripheral blood of MM patients compared with healthy people and correlated with disease activity, but no significant change was found in bone marrow. The percentage of nTregs in MM peripheral blood was higher than that in the corresponding bone marrow samples. There was no significant correlation between IL-10 and TGF-β levels and the number of nTregs. The percentage of double-negative Tregs in MM peripheral blood was decreased and the degree of reduction was negatively correlated with the disease burden [87]. Muthu et al. [88] studied changes in CD8^+^Treg cells in MM: The frequency and absolute number of CD8^+^CD25^high^Foxp3^+^ Treg cells in MM patients were significantly higher than those in healthy people, and the expression level of CTLA-4 was increased. There was a negative correlation between the number of CD8^+^Treg cells and the total lymphocytes from peripheral blood and bone marrow. In addition, many studies have investigated the changes in the number and function of Treg cells in MM. Different studies have shown different trends in the changes of Treg cells in the peripheral or bone marrow of MM, with a decrease [89, 90], an increase [20, 77, 91–95], or no significant change [96–98].

#### Distribution of Treg cells in peripheral blood and bone marrow of MM

The distribution of Tregs in bone marrow and peripheral blood is also controversial. It has been proposed that in normal bone marrow, 70% of CD4^+^CD25^+^ cells express Foxp3, compared with 30% in peripheral blood of the same person. In contrast, MM patients expressed Foxp3 on only 2.2% of CD4^+^CD25^+^ cells in the bone marrow, while 52.2% in the peripheral blood. This is because the cytokine profile in the bone marrow of MM patients tends to promote and maintain the proliferation of Th17 cells, making the myeloma microenvironment markedly deficient in Tregs [99]. Another study did not find any difference in the distribution of total Treg cells in bone marrow and peripheral blood, but suggested that specific subsets (including effector/effector memory Tregs, terminal effector Tregs and CD39^+^ Tregs) with enhanced suppressive function were prevalent in bone marrow tumor sites [100]. Guichelaar et al. [101] investigated whether MM bone marrow stromal could cause Treg cell transformation in vitro. The suppressive capacity of Tregs cultured in bone marrow stromal cells was significantly reduced, without significant reduction in the Foxp3 expression. And the proportion of Foxp3^+^ T cells was increased, demonstrating IL-17 production. Neutralization of IL-1b and IL-6 activity in the culture medium was able to restore the suppressive activity of Treg cells in some samples.

#### Correlation between Treg cells and clinical features of MM

The level of Tregs is correlated with the clinical characteristics of MM patients. Hypercalcemia, malignant plasma cell level, IgA subtype, lactate dehydrogenase and β2 microglobulin levels were all associated with Tregs level. A higher frequency of Tregs in peripheral blood was associated with shorter time to progression (TTP), shorter survival, worse treatment response and susceptibility to infection [91, 92, 94, 102, 103]. Studies on long-term survival of MM patients have found that the frequency and absolute count of Treg cells were significantly lower, the number of Th17 cells was increased, and the Treg/Th17 ratio was decreased compared with ordinary MM patients [104]. In addition, Tregs were found to be increased in the peripheral blood of patients with high-risk SMM [105], further indicating the relationship between Tregs and the prognosis and disease progression of MM. However, in the study by Foglietta et al. [96], the total number, phenotype, function, and TCR diversity of Tregs were not affected by disease status and were not clearly correlated with the pattern of myeloma cell infiltration. It was also believed that the absolute number and proportion of Tregs or its subsets can not predict the prognosis of MM and the transformation of MGUS [97, 106]. Adenosine is a growth factor for osteoblasts and osteoclasts, so the CD39/CD73 adenosine pathway is also particularly important for the pathogenesis of MM. The prevalence of CD39^-^Treg cells was observed in MM, which was speculated to be related to the production of IL-17 and the progression of MGUS to MM [107].

### Regulatory T cells and treatment options for MM

At present, there are many treatment options for multiple myeloma, including IMiDs, PIs, mAbs, cytotoxic drugs, and HSCT. Depending on the mechanism of action, these treatments may cause different changes in the level and function of Tregs, which may affect the therapeutic effect (Table 2).

**Table 2:** Changes of regulatory T cells and treatment options for multiple myeloma.

treatment options		Subject	Number	Function	Expression of Foxp3	Gating method	Reference
IMiDs	thalidomide	cell	ns		ns	Foxp3+CTLA-4highCD25highCD4+	[108]
		cell	↑	↓		CD4+Foxp3+	[112]
		mice	ns			CD4+Foxp3+	[112]
		patients	↓			CD4+CD25+	[114]
		patients	↑			CD4+CD25+Foxp3+	[87]
		patients	↑			CD4+CD25+CD127lowFoxp3+	[90]
		patients	↑			CD127dimCD25highFoxp3+	[115]
	lenalidomide	cell	↓	↓	↓	Foxp3+CTLA-4highCD25highCD4+	[108]
		cell		↓		CD4+CD25highCD127low	[109]
		cell	↓			CD4+CD25+Foxp3+	[111]
		mice	↓			CD4+Foxp3+	[113]
		patients	↓*			CD4+CD25highCD127neg/lowFoxp3+	[122]
		patients	↑*			CD8+CD25highFoxp3+	[116]
		patients	↑*			CD4+CD25+CD127lowFoxp3+	[105]
		patients	↑			CD45RA-CD4+Foxp3high	[117]
	pomalidomide	cell	↓	↓	↓	Foxp3+CTLA-4highCD25highCD4+	[108]
		cell		↓		CD4+CD25highCD127low	[109]
PIs	bortezomib	patients	ns			CD4+CD25highCD127neg/lowFoxp3+	[122]
		patients	ns			CD4+CD25+Foxp3+	[123]
mAbs	daratumumab	patients	↓			CD4+CD25+CD127dim	[126]
		patients	↓			CD4+CD25+CD127dim	[127]
		patients	ns			CD4+CD25+CD127dim	[129]
		patients	↓			CD4+CD25+CD127dim	[128]
		patients	↓			CD45+CD4+CD25highCD127dim	[130]
	Isatuximab	cell	↓	↓		CD4+CD25highFoxp3+	[20]
	anti-CD137 antibody	mice	↑			CD4+Foxp3+	[133]
	elotuzumab	patients	↓			CD3+CD8+CD28-CD57+	[135]
	anti-PD-1 antibody	patients	↑			CD3+CD4+/Foxp3+CD25+	[136]
	anti-TIGIT antibody	cell		↓		CD4+CD25+CD127-Foxp3+	[137]
cytotoxic drugs	cyclophosphamide	patients	ns†			CD4+CD25high	[138]
		patients	↓↑				[140]
		mice	↓				[113, 139]
HSCT	ASCT	patients	↑			CD4+CD25+Foxp3+	[141]
		patients	↓			CD3+CD4+CD25brightCD127neg	[142]
		patients	↑			CD4+Foxp3+	[143]
	Allo-HSCT	patients	↑			CD4+Foxp3+	[146]
		patients	ns			CD4+CD25_CD127-/low	[147]

*↑* Increased number or enhanced functionality, ↓ reduced quantity or inhibited function, *ns* no significant difference.

^†^Combined treatment with G-CSF.

*Combined therapy with dexamethasone.

#### Tregs and IMiDs

IMiDs can induce the expression of tumor suppressor genes, cell cycle arrest, caspase activation and cell apoptosis in MM cell lines by acting on myeloma cells and bone marrow hematopoietic microenvironment. It plays an immunomodulatory role while killing tumor cells, and enhances the activity of CD8^+^ T cells, NK cells and NKT cells. In the in vitro study, PBMCs were treated with IMiDs (thalidomide, lenalidomide and pomalidomide) for 7 days, and Foxp3^+^CTLA-4^high^CD25^high^CD4^+^ cells were detected. The results showed that the proportion of Tregs in thalidomide group did not change significantly, while the other two groups significantly decreased [108]. De Keersmaecker et al. [109] co-cultured CD4^+^CD25^high^CD127^low^ Tregs isolated by flow cytometry with CD8^+^ cytotoxic T cells and treated with lenalidomide or pomalidomide. They found that the ability of treated Treg to inhibit CD8^+^ T cell proliferation and cytokine production was decreased. This might be based on a decrease in Foxp3 expression [110]. In addition, lenalidomide could abolish the ability of human-derived MM cells to induce Treg cells in vitro [111]. Being different from these results, Kim et al. [112] observed increased differentiation of Tregs after treatment of naive T cells with thalidomide. In animal studies, treatment of MM tumor-bearing mice with lenalidomide was found to reduce Tregs’ level in the spleen [113]. In clinical studies, Yang et al. [114] compared the changes of CD4^+^CD25^+^ Tregs in MM patients before and after thalidomide application, and proposed that thalidomide may play an anti-MM effect by down-regulating Tregs. However, other studies have shown the opposite results, with an increase in Tregs observed during thalidomide treatment relative to baseline [87, 90, 115]. Treatment with lenalidomide in newly diagnosed MM patients could increase the level of CD8^+^ Treg cells [116]. Tregs increased in high-risk SMM patients treated with lenalidomide and dexamethasone [105], and similar results were observed in MM patients treated with lenalidomide maintenance therapy [117]. Some researchers believed that the increase of Tregs was an indicator of disease control in MM patients, and others believed that it could affect the therapeutic effect of IMiDs in MM. These inconsistent results may be due to a variety of factors, such as different choices of Treg gating, differences in the duration of IMiDs application, and different time points of sample collection.

#### Tregs and PIs

The proteasome plays an important role in the regulation of a variety of signaling pathways and the degradation of proteins. The proliferation of myeloma cells is dependent on proteasome-regulated signaling pathways, and thus they are susceptible to PIs [118]. PIs can selectively bind to the active site of the proteasome, inhibit chymotrypsin activity, and promote myeloma cell apoptosis by reducing cytokine expression levels, thereby preventing tumor growth, spread, and angiogenesis. In other diseases and their models or in vitro, the use of PI bortezomib can increase Tregs level and maintain Foxp3 expression [119–121]. There were few related studies in MM. A clinical study showed that there was no significant change in the frequency of Tregs during the treatment of MM patients with bortezomib and dexamethasone [122]. Similar results were seen in Shi et al. [123], where bortezomib could reverse the immunosuppressive state of MM without affecting the Treg cell count. Ercetin et al. [124] explored the role of Tregs in the treatment of MM with bortezomib. Tregs were collected from the peripheral blood of newly diagnosed MM patients and expanded. Myeloma cells were treated with bortezomib alone or in combination with expanded Tregs in vitro. The results showed that Tregs combined with bortezomib killed more malignant myeloma cells than bortezomib alone. They also monitored the clinical status of the patients and found a statistically significant correlation between bortezomib sensitivity and Tregs expansion [124].

#### Tregs and mAbs

Monoclonal antibodies are produced by the fusion of immune B cells that can make antibodies against specific antigenic determinants with myeloma cells. Daratumumab, the first mAb approved for MM, targets CD38 and induces tumor cell death through multiple mechanisms, including antibody-dependent cell-mediated cytotoxicity (ADCC), complement-dependent cytotoxicity (ADC), and antibody-dependent cellular phagocytosis (ADCP) [125]. Multiple studies have found that a small fraction of CD4^+^ Treg cells in MM patients expressed high levels of CD38 before activation. These cells were more potent than CD38^-^Treg cells and were highly sensitive to daratumumab, showing a significant decrease in number after daratumumab treatment [126–129]. The number of CD38^+^ Treg cells in relapsed/refractory MM patients was significantly higher than that in non-relapsed/refractory MM patients and healthy controls [130]. The application of daratumumab significantly reduced the absolute number of Tregs in these patients. Moreover, the frequency of circulating CD38^+^ Tregs before treatment was related to the degree of response to daratumumab, and patients with high cell frequency showed durable responses, while the number of CD38^-^ Tregs did not change significantly during treatment [130]. Deep sustained responses to daratumumab monotherapy were associated with a substantial reduction in Treg frequency in high-risk MM patients [131]. The proportion of CD38^+^ cells in Tregs was higher than that in effector T cells, and the level of CD38 was correlated with the expression level of Foxp3 [20]. Isatuximab, also a monoclonal antibody against CD38, reduced the number of CD38-overexpressing Treg cells and the production of immunosuppressive factors, and the inhibition was CD38-specific and Fc independent [20]. CD137 is a costimulatory molecule expressed on activated T cells, NK cells, antigen presenting cells, and endothelial cells. Agonistic anti-CD137 antibody can exert anti-MM effects dependent on the activation of the classical NF-κB pathway [132]. In the MM mouse model, anti-CD137 mAb injection induced proliferation of Tregs at an early stage, and this proliferation may limited the antineoplastic effect [133]. SLAMF7, a glycoprotein expressed on the surface of myeloma cells, is also present on the surface of NK cells and plasma cells, and is a strong marker of normal and malignant plasma cells in MM. CD3^+^CD8^+^CD28^-^CD57^+^ T cells are a subset of CD8^+^ Treg cells, which exert immunosuppressive effects through soluble factors in a non-antigen specificity manner [134]. Awwad et al. [135] found that SLAMF7 was highly expressed by this cell subset in MM patients, and a significant reduction in the percentage of CD8^+^ Tregs was observed after treatment with the anti-SLAMF7 mAb elotuzumab. Programmed death receptor 1 (PD-1) is an inhibitory immune checkpoint, which promotes self-tolerance by downregulating the response of the immune system to self cells and inhibiting the inflammatory activity of T cells. Myeloma cells can achieve immune escape by expressing PD-1 ligands. In a study published in *Leukemia* [136], increased PD-1 expression was observed in CD4^+^ Treg cells from bone marrow and peripheral blood of MM patients, whereas treatment with anti-PD-1 antibody induced expansion of Tregs in bone marrow mononuclear cells (BMMC). TIGIT is a co-inhibitory receptor highly expressed in tumor-infiltrating lymphocytes, which is a new immune checkpoint after PD-1/PD-L1. TIGIT is highly expressed on the surface of most Tregs in humans, which can enhance their immunosuppressive function and stability. Jeong et al. [137] developed an IgG4-type mAb against human TIGIT, MG1131, which could bind to cell surface TIGIT with high affinity and enhance NK cell-mediated tumor killing activity. Moreover, it could inhibit the activity of Tregs in vitro and reduce the expression of inhibitory immune checkpoint molecules CTLA-4, CD39 and PD-1. In conclusion, different mAbs may have different effects on the number and function of Treg cells in MM patients due to their different specific targets and mechanisms of action, which in turn affects the anti-myeloma effect of antibody drugs.

#### Tregs and cytotoxic drugs

Cyclophosphamide is the most commonly used alkylating agent class of antitumor drugs, cell cycle non-specific cytotoxic drugs. After entry into the body, cyclophosphamide is decomposed into phosphoramide nitrogen mustard under the catalysis of liver microsomal enzyme, resulting in cytotoxic effect on tumor cells and immunosuppressive effect. Cyclophosphamide is widely used for induction chemotherapy, mobilization of autologous hematopoietic stem cells, and autologous stem cell transplantation (ASCT) in MM. One study showed that the combination of high-dose cyclophosphamide and granulocyte colony-stimulating factor (G-CSF) resulted in a severe decrease in the number of T cells, but the recovery rate of Tregs was higher than that of other lymphocyte subsets, so there was no loss of Tregs [138]. In a mouse model of MM, low doses of cyclophosphamide could cause selective and transient depletion of Tregs, resulting in the proliferation of effector T cells in peripheral blood and the recovery of immune function [139]. In some patients, repeated low doses of cyclophosphamide can also reduce Treg cell levels for several days per dose, although Tregs tend to increase in the long term [140]. However, this effect may not be stable due to the large interindividual differences in cyclophosphamide pharmacokinetics.

#### Tregs and HSCT in MM

In the treatment of MM, ASCT is preferred after effective induction therapy for patients with good performance status, but it still cannot prevent disease progression and relapse. The reconstitutions of immune cell subsets after transplantation are particularly important for the outcome of immunotherapy. The correlation between ASCT and Treg cell level has been studied. Patients’ peripheral blood was collected between 40 and 110 days after transplantation. The percentage of Tregs in patients who received transplantation was significantly higher than that in patients who did not receive transplantation. MM patients with higher Treg frequency after transplantation had a longer survival time than those with lower Treg frequency [141]. Another study, published in 2016, addressed the process of immune reconstitution [142]. In this prospective clinical trial, patients underwent ASCT followed by lenalidomide maintenance therapy. Peripheral blood samples were collected at various time points to monitor disease status. The ratio of Tregs /CD8+ effector T cells decreased significantly 12 days after transplantation, and then gradually recovered and reached the peak at 30 days after transplantation. This provided a valuable window for immune consolidation therapy after transplantation. The researchers also found that the increase in Tregs after transplantation might be related to disease recurrence [142]. Lee et al. [143] examined the T-cell phenotype of bone marrow infiltrating lymphocytes in patients at day 100 after ASCT to explore its association with disease-free survival. Bone marrow Tregs in MM patients had a higher frequency and expressed higher levels of ICOS, CTLA-4, and CD25 after ASCT compared with healthy controls. In addition, the frequency of Tregs in MM patients was not significantly correlated with the length of progression-free survival (PFS) after transplantation, which was inconsistent with the conclusion of previous studies [141].

In the guideline, it is mentioned that allogeneic HSCT (Allo-HSCT) can be considered for young newly diagnosed MM patients with high-risk prognostic factors and suitable donors or relapsed patients. It has been reported that the relapse rate of MM decreases after Allo-HSCT treatment [144, 145]. However, due to scruples about transplantation-related toxic effects and the development of various treatment options such as PIs, IMiDs, and mAbs, Allo-HSCT is not widely used in clinical practice. Because high-dose chemotherapy destroys the thymus in patients with MM undergoing HSCT, expansion of Tregs in these patients can only be based on donor-derived Tregs or differentiation of conventional T cells. Atanackovic et al. [146] used flow cytometry to detect the percentage of CD4^+^Foxp3^+^ Tregs in the bone marrow of MM patients after Allo-HSCT. The results showed that Tregs experienced a significant expansion after transplantation, and the progression or relapse of MM did not have any effect on the number of Tregs. However, a study published in 2015 observed an increase in the frequency of Tregs in MM patients with disease progression after Allo-HSCT, and the increase of Tregs was associated with shorter PFS and Overall Survival (OS) [147].

### Application of Tregs in the treatment of MM

It is currently accepted that Tregs in MM suppress antitumor immunity, and reducing the number of Tregs or inhibiting their function may have therapeutic effects in MM. B7-H1 is a PD-1 ligand (PD-L1). B7-H1 in TME participates in the induction of CD4^+^Foxp3^+^ Treg through B7-H1/PD-1 signaling pathway. Zhang et al. [148] constructed rhB7-H1M protein vaccine and inoculated it into myeloma mouse models, and observed that tumor growth was significantly inhibited and the proportion of Tregs in peripheral blood of mice was significantly reduced. Kawano [14] used Treg knockout mice (DEREG) to construct MM model and used diphtheria toxin to deplete Tregs. Compared with wild-type mice + diphtheria toxin treatment group and Treg knockout mice +PBS treatment group, the survival time was significantly prolonged. A recent study also demonstrated similar results: CD4^+^ and CD8^+^ T cell maturation and activation were increased, the proportion of NK cells was increased, the killing effect was enhanced, and MM disease was alleviated after Treg depletion in a MM mouse model. The short-term reduction of Tregs was enough to achieve long-term and stable remission of MM [81]. Effector Tregs are the most abundant cell type among Foxp3^+^ T cells in tumor tissues [149], and the selection of their surface specific targets is expected to activate antitumor immunity while retaining other Tregs that are essential for suppressing autoimmunity [150]. Some studies have focused on how to design anti-CD25 antibody drugs to selectively deplete Tregs without affecting the function of effector T cells, but their use in clinical practice has not achieved ideal results. Vargas et al. found that the widely used anti-CD25 antibody (PC-61, αCD25-r1) almost exclusively kills peripheral Tregs and does not effectively inhibit tumor infiltrating Tregs because of high intra-tumoral expression of FcγRIIb. By modifying the anti-CD25-r1 to efficiently deplete intra-tumoral Tregs, the team opened up a new ground for the use of CD25 antibody drugs [151]. Derman et al. then evaluated the feasibility and efficacy of in vivo Treg depletion (IVTRD) and ex vivo Treg depletion (EVTRD) in the treatment of MM HSCT. IVTRD referred to the use of anti-CD25 mAb to reduce the frequency of Tregs in MM patients, while EVTRD referred to the isolation and removal of Tregs from the graft before transplantation. The results showed that patients in the IVTRD and EVTRD groups had a better response, a higher 3-year PFS rate and no significant autoimmune complications compared with those in the control group [152].

Due to the immunosuppressive effect of Tregs, their potential for preventing graft-versus-host disease (GVHD) after HSCT in MM has been extensively explored. Studies have shown that deficiency of Tregs after Allo-HSCT is associated with severe acute GVHD [153], proliferation of Tregs and activation of inhibitory functions can reduce the severity of GVHD [154]. The U266 myeloma cell line is highly immunogenic and grows exclusively in the bone marrow, whereas the LME-1 cell line can grow both in the bone marrow and extramedullary. Guichelaar et al. [101] used the above cell lines with different characteristics to establish MM mouse models. Human PBMC were infused into the MM model of U266 to mimic immune system transplantation. Although the tumor was eliminated well, these mice died of severe GVHD. In addition to human PBMC, co-infusion of ex vivo expanded Tregs significantly inhibited lethal GVDH without affecting graft-versus-tumor effect (GVT). Using the same method, the LME-1 cell line showed different results: Co-infusion of Tregs and PBMC led to significant growth of extramedullary tumors, while the effect of GVT on intramedullary tumors was basically not affected [101]. These results offered the possibility of exogenous Tregs infusion for prevention GVHD in the context of hematologic malignancies progressing entirely in the bone marrow.

## Conclusions

Recent advances in cancer biology have shown that TME plays an important role in the occurrence and development of malignant tumors. MM, as a hematological malignancy, mainly exists in the bone marrow, and also has extramedullary invasion. The suppressive immune microenvironment composed of various immune cells in bone marrow and peripheral blood has been widely studied. Tregs play a central role in immune suppression. It has been recognized that Tregs increase in number and function in a variety of solid tumors. However, due to the complexity of MM bone marrow microenvironment and the disunity of Treg gating methods, the changes in the number and function of Tregs in MM are still controversial. At present, the researches on Tregs in MM are mostly descriptions of the phenomenon, and the exploration of its regulatory mechanism is still not deep. The effect of various treatment modalities on Tregs and the relationship between Treg levels and MM prognosis are also unclear. It is certain that reducing the number or inhibiting the function of Tregs is beneficial to the recovery of anti-tumor immunity, but it is necessary to master the balance between immune suppression and immune overactivation to avoid the occurrence of autoimmune diseases. In summary, Tregs provide a new pointcut for the treatment of MM.
